# Genomics of hybrid parallel origin in *Aquilegia ecalcarata*

**DOI:** 10.1186/s12862-024-02266-7

**Published:** 2024-06-06

**Authors:** Fang-Dong Geng, Miao-Qing Liu, Xue-Dong Zhang, Lu-Zhen Wang, Meng-Fan Lei

**Affiliations:** 1 Key Laboratory of Resource Biology and Biotechnology in Western China, Ministry of Education, College of Life Sciences, Northwest University, Xi’an, China; 2 College of Life Sciences, Shaanxi Normal University, Xi’an, China

**Keywords:** *Aquilegia Ecalcarata*, Gene flow, Highly diverged regions, Parallel evolution

## Abstract

**Background:**

The parallel evolution of similar traits or species provides strong evidence for the role of natural selection in evolution. Traits or species that evolved repeatedly can be driven by separate de novo mutations or interspecific gene flow. Although parallel evolution has been reported in many studies, documented cases of parallel evolution caused by gene flow are scarce by comparison. *Aquilegia ecalcarata* and *A. kansuensis* belong to the genus of *Aquilegia*, and are the closest related sister species. Mutiple origins of *A. ecalcarata* have been reported in previous studies, but whether they have been driven by separate de novo mutations or gene flow remains unclear.

**Results:**

In this study, We conducted genomic analysis from 158 individuals of two repeatedly evolving pairs of *A. ecalcarata* and *A. kansuensis*. All samples were divided into two distinct clades with obvious geographical distribution based on phylogeny and population structure. Demographic modeling revealed that the origin of the *A. ecalcarata* in the Eastern of China was caused by gene flow, and the Eastern *A. ecalcarata* occurred following introgression from Western *A. ecalcarata* population. Analysis of Treemix and *D*-statistic also revealed that a strong signal of gene flow was detected from Western *A. ecalcarata* to Eastern *A. ecalcarata.* Genetic divergence and selective sweep analyses inferred parallel regions of genomic divergence and identified many candidate genes associated with ecologically adaptive divergence between species pair. Comparative analysis of parallel diverged regions and gene introgression confirms that gene flow contributed to the parallel evolution of *A. ecalcarata*.

**Conclusions:**

Our results further confirmed the multiple origins of *A. ecalcarata* and highlighted the roles of gene flow. These findings provide new evidence for parallel origin after hybridization as well as insights into the ecological adaptation mechanisms underlying the parallel origins of species.

**Supplementary Information:**

The online version contains supplementary material available at 10.1186/s12862-024-02266-7.

## Introduction

Independent populations that colonize similar environments and evolve similar traits provide strong evidence for the deterministic role of natural selection in evolution. The resulting pattern has been called “parallel” or “convergent” [1–5]. Replication occurs at different levels of biological tissue, including genes, pathways, networks, univariate and multivariate phenotypes, ecological traits, and biological communities, and may lead to replicated evolution of species or ecotypes [6–9]. Indeed, the genetic mechanisms underlying parallel evolution are often unclear in many studies of repeated evolution. Nevertheless, the traits that evolved repeatedly in parallel are often assumed to have arisen independently through separate de novo mutations (narrow-sense definition of parallel evolution), but such mutations could also have been recruited from shared ancestral polymorphisms or interspecific gene flow [10–12]. For example, widespread parallel evolution in sticklebacks was due to repeated fixation of ectodysplasin alleles [13], and Pundamilia cichlid species appeared after hybridization [14, 15].

With the advent of population genomic data, it is now possible to detect genomic regions putatively underlying recent convergent adaptations. Introgression hybridization has been proposed as an essential source of adaptive genetic variation [16]. Soria-Carrasco et al. [17] found that 17% of the single-nucleotide polymorphisms (SNPs) in the genome of *Timema cristinae* in California occurred between two or more pairs of parallel ecotypes and that 0.01% of SNPs were affected by natural selection according to a field experiment. Meier et al. [15] used genomic analyses to study the parallel ecological speciation of blue and red-backed *Pundamilia cichlid* species in Lake Victoria. Their findings revealed that a subset of the most strongly diverged regions in older species pairs also diverged in younger pairs, and these shared diverged regions exhibited parallel differences in allele frequency.

Parallel origins of *Aquilegia ecalcarata* have been documented by previous studies [18, 19]. *A. ecalcarata* has been divided into the Eastern clade and Western clade in China based on population genomic data. The Eastern clade includes *A. ecalcarata* and *A. kansuensis*, and the Western clade includes *A. ecalcarata*, *A. rockii*, and *A. kansuensis*. The genetic introgression from Western *A. ecalcarata* has contributed to the emergence of the *A. rockii* phenotype with straight and short nectar spur [19]. The main morphological differences between *A. ecalcarata* and *A. kansuensis* include the size of the flower organs and the presence or absence of nectar spurs, *A. kansuensis* has nectar spurs and the nectar spurs have played a key role in the floral isolation between *A. ecalcarata* and *A. kansuensis* [19]. The multiple origin of *A. ecalcarata* are adapted to a stony environment that differs from that of their closest relatives, indicating a habitat shift may have driven new adaptations [18]. Ballerini et al. [20] found that the *POPOVICH* plays a critically important role in nectar spur development and has recently been shown to encodes a *C2H2* zinc-finger transcription factor. It has been reported that *POPOVICH* plays a central role in regulating cell proliferation in the *Aquilegia* petal during the early phase of spur development [20]. The *POPOVICH* gene is located on linkage chromosome3_27454200–27,455,760 in the *A. coerulea* ‘Goldsmith’ v3.1 reference genome (https://phytozome.jgi.doe.gov) corresponding to linkage chromosome3_26779918–26,781,011 of the *A. oxysepala* var. *kansuensis* v1.0 genome assembly [21]. Geng et al. [19] analyzed the shared alleles of the *POPOVICH* gene in *A. ecalcarata* lineages of different origins and found that the spurless trait might have a single origin. However, incomplete lineage sorting precluded determination of whether the different *A. ecalcarata* lineages originated through hybridization. Thus, whether the different origins of *A. ecalcarata* lineages were driven by multiple independent mutational or introgression hybridization remain unclear.

Here, we conducted a genomic analysis of 158 individuals of two repeatedly evolving pairs (Eastern species pair and Western species pair) of *A. ecalcarata* and *A. kansuensis*. Using population genetic structure, model simulations, treemix and ABBA-BABA tests, genetic differentiation, GO enrichment analysis and patterns of isolation by distance (IBD), determine the origin pattern of Eastern and Western *A. ecalcarata*, detect the important role of gene flow in parallel evolution, and infer the genetic basis of multiple origin of *A. ecalcarata*.

## Materials and methods

### Sample collection, sequencing, variant calling and filtering

A total of 158 individuals were selected for genomic analysis, populations information was shown in Table S1. The first author identifed and deposited voucher specimens in the herbarium of Shaanxi Normal University (SNNU) (Table S1). All specimens were collected from the wild. Eastern species pair (including 40 *A. kansuensis* and 55 *A. ecalcarata*) and Western species pair (including 23 *A. kansuensis* and 40 *A. ecalcarata*). DNA was extracted from dried leaves using a Genomic DNA extraction kit. Libraries were generated using the Illumina Next Ultra DNA Library Prep Kit (TIANGEN, BEIJING, China). Sequencing libraries were sequenced on the Illumina HiSeq 4000 platform (San Diego, CA, USA) to generate 150 bp paired reads with the sequencing depth of 20×. All obtained DNA reads have been deposited in the NCBI database under BioProject: PRJNA690975, and individual number information was shown in Table S1. High quality clean reads were mapped to *A. yangii* reference genome [21] using bwa v0.7.15 with default options [22]. Alignment results and marked duplicate reads were sorted using samtools v1.3.1 [23], and duplicate reads were removed using samtools v1.3.1. Variants were called using samtools v1.3.1 and filtered using VCFtools v0.1.13 [24]. The specific commands and parameters used were as follows: Samtools calling (multisample): samtools mpileup -b bam.list ‐C 50 ‐q 25 ‐f ‐‐output ‐v ‐u ‐t DP ‐t AD ‐t SP ‐e ‐h ‐L ‐o –p. Vcftools filtering: vcftools –vcf ‐‐minQ 25 –min‐meanDP 5 ‐‐max‐‐meanDP 30 ‐‐maf 0.02 ‐‐max‐missing 0.5 –out.

### Phylogenetic inference

We converted the vcf file into a fasta file using a perl script. The script handled the loci as follows: replacing heterozygous loci with AC = > M, CA = > M, AG = > R, GA = > R,AT = > W, TA = > W, CG = > S, GC = > S, CT = > Y, TC = > Y, TG = > K, and GT = > K, and all non-variant sites were removed. We regarded *A. yabeana* (The name of the three individuals: NM0101, BJ0101 and HA0101, Table S1) as outgroup and constructed the maximum likelihood (ML) tree using IQ-TREE v2.0.3 [25] under the GTR model [26] with 1,000 bootstrap replicates [27]. The phylogenetic tree was edited and modified using FigTree v1.4.4 (http://tree.bio.ed.ac.uk/software/figtree/).

### Genetic structure

The population genetic structure in our samples was inferred using ADMIXTURE v.1.4.0 [28]. The postulated number of ancestral populations (*K*) was set from 2 to 8, and 10-fold cross-validation (--cv = 10) was performed. We selected the most likely *K* based on the minimum cross-validation (CV) error. Principal components analysis (PCA), a dimensionality-reduction method, was conducted to further assess the population structure. First, we converted the format using VCFtools v0.1.16 and PLINK v1.9 [29]. Then, SNPs were filtered with the parameters as “--indep-pairwise 50 5 0.5”. PCA was accomplished on all SNPs using smartpca program from EIGENSOFT v6.1.4 [30] with default parameters.

### Demographic history

To discriminate among alternative evolutionary scenarios for the origin of the *A. ecalcarata* and *A. kansuensis*, we used fastsimcoal2 v2.6 [31] to conduct model simulations. First, in order to assess whether speciation occurred in a period of geographical isolation or in the face of gene flow, we conducted model simulations for both Eastern and Western species pair. To test whether the divergence of *A. ecalcarata* and *A. kansuensis* was accompanied gene flow, we compared four demographic models for the Eastern and Western pairs: (1) no gene flow; (2) secondary contact (only recent gene flow); (3) only early gene flow; (4) constant gene flow. The change time in gene flow was estimated as a model parameter (Table S2). Next, we constructed alternative demographic models for the evolution of Eastern and Western species pair combined. We compared six topologically different demographic models: (1) a single origin model, wherein Eastern *A. kansuensis* first diverged from ancestral group, followed by Western *A. kansuensis*, Eastern and Western *A. ecalcarata* eventually diverged, allowing for recent gene flow between species pairs and between the same species in Eastern and Western populations; (2) a single origin model, wherein Western *A. kansuensis* first diverged from ancestral group, followed by Eastern *A. kansuensis*, Eastern and Western *A. ecalcarata* eventually diverged, allowing for recent gene flow between species pairs and between the same species in Eastern and Western populations; (3) a single origin between *A. kansuensis* and *A. ecalcarata* with subsequent independent colonization of the West and the East by both species and interspecific gene flow and conspecific gene flow between species in Eastern and Western populations; (4) a parallel origin with two independent evolution events into *A. kansuensis* and *A. ecalcarata*, wherein one species pair occurs in the East, and the other one in the West, allowing for subsequent gene flow between species pairs and between the same species in Eastern and Western populations; (5) a hybrid parallel origin (paralle origin after hybridization) model, wherein the Western species pair is derived from a hybrid ancestor, allowing for recent gene flow between species pairs and between the same species in Eastern and Western populations; and (6) a hybrid parallel origin model, wherein the Eastern species pair is derived from a hybrid ancestor, allowing for recent gene flow between species pairs andas well as between the same species in Eastern and Western populations. The estimated generation time were set to 2 year and mutation rata were set to 7e−9 per base pair per generation based on the rate of *Arabidopsis thaliana* [32]. Alternative models of historical events were fitted to the joint site frequency spectra data, and two-dimensional joint SFS (2D-SFS) was constructed from posterior probabilities of sample allele frequencies using easySFS.py (https://github.com/isaacovercast/easySFS). Each model was run 50 times with 100,000 simulations to calculate composite likelihood and 40 expectation-conditional maximization (ECM) cycles. The best model was identified using the maximum likelihoods value distributions and Akaike’s information criterion (AIC) [31]. Finally, we calculated 95% confidence intervals of demographic parameters estimated from 100 bootstrap replicates by simulating SFS from the maximum composite likelihood estimates and re-estimating parameters. To further verify the results of fastsimcoal2, we used Migrate-n software to infer gene flow between the Eastern and Western *A. ecalcarata* [33]. Six models were used to infer different patterns of gene flow: (1) Western *A. ecalcarata* had a past gene flow to Eastern *A. ecalcarata*, (2) Eastern *A. ecalcarata* had a past gene flow to Western *A. ecalcarata*, (3) there was bidirectional gene flow between Eastern and Western *A. ecalcarata*, (4) there was no past gene flow between Eastern and Western *A. ecalcarata*, (5) Eastern *A. ecalcarata* had a past gene flow to the ancestral population of Western group, and (6) Western *A. ecalcarata* had a past gene flow to the ancestral population of Eastern group. The specific parameters used were: ML analysis strategy, 10 short chains (totaling 10,000 trees) and 3 long chains (totaling 500,000 trees), burn-in of the initial 100,000 trees, adaptive heating scheme (heating = ADAPTIVE), four temperature intervals of 1, 1.2, 1.5, and 3, with other settings using default parameters. The best model was determined using the maximum likelihood value.

We executed pairwise sequentially Markovian coalescent (PSMC) modeling [22] to estimate historical changes in *Ne* (effective population size) through periods based on each species. The *Ne* was also calculated using SMC + + v. 1.15.2. The mutation rate was 1.4E-8 per site per year, and the one generation was 2 years [19].

### Detection of gene flow

Treemix v1.13 [34] was used to infer the direction and strength of gene flow. The number of migration edges (m parameter) of Treemix algorithm was set from 2 to 5, and per m parameter was iterated 5 times. The default “Evanno”-like method and various linear model in R package OptM [35] were used to determine the best m value. To further confirm the genetic introgression, ABBA-BABA analysis (*D*-statistic) was conducted with Dsuite v0.3 [36]. The *D*-statistic [37] is designed for a 4-taxon fixed phylogeny of P1, P2, P3, and outgroup (O). When there is no gene introgression between O, P1, P2 and P3, the *D*-statistic would be 0. If, however, there is gene introgression between P2 and P3 or P1 and P3, the *D*-statistic would be positive or negative. The vcf file was used directly as the input file, and SNPs were filtered using parameters “--maf 0.05 --geno 0.2”. The Dtrios procedure was then used to identify gene introgression at a significant level of *p* < 0.01. In addition, we used *fd* statistics to more accurately analyze the gene flow between different originated *A. ecalcarata*, and we used 5 kb window to analyze *fd* statistics [38].

### Genetic divergence and population selection

In order to study genetic divergence and population selection among species, genome scanning was used to analyze the genomic data, and all statistics were used a nonoverlapping 5Kb window. The relative divergence (*Fst*), the absolute divergence (*Dxy*) and the nucleotide polymorphism (*π*) were calculated using the Python script [38]. The population selection tests was performed through XP-CLR analysis [39] with nonoverlapping 5Kb window, linkage disequilibeium (LD) value is 0.95, and the minimum number of SNPs in each window is set to 200. Statistical analysis was conducted on the Eastern and Western species pairs, respectively. We standardized per-window *Fst* or *Dxy* in each group pair to a Z-score [40] based on the formula: Z-value = (window value − mean value)/standard deviation value. The windows with Z-FST ≥ 2 or Z-*Dxy* ≥ 2 from each species pair were treated as highly diverged regions (HDRs) [41]. Venn diagram was calculated and visualised using the jvenn online website (http://bioinfo.genotoul.fr/jvenn). Ggplot was used to visualize the boxplot, ggsignif is used to add significance markers, significance test was performed using *t-test* (10.31234/osf.io/7awm6).

To further demonstrate the impact of gene introgression on genetic divergence in Eastern and Western specie, we then calculated and compared the genomic characteristics of the gene introgression regions, and we used “bedtools shuffle” to randomly generate 1000 genomic regions, each of which was the same size, so that these genomic regions were representative of the genome-wide range. We compared the *Fst* and *Dxy* between the introgression region and 1,000 randomly selected genomic regions with same size.

### Gene Ontology (GO) annotation

We selected the target regions on the genome and bcftools v1.1 [23] was used to export the fasta sequence, which then imported into the OmicsBox software (http://manual.omicsbox.com). Blast analysis was used, followed by running InterproScan and merging InterproScan GOs to annotation. GO annotation was performed after GO mapping with default parameters. GO-weight was set to 5, e-value-hit-filter to 1e-3, and other parameters to default values.

### Haplotype analysis and isolation by distance (IBD)

Haplotype were estimated with ape and pegas in R v3.4.3, and network were drawn through PopART v1.7 [42]. Then, pheatmap in R v3.4.3 was used to draw the haplotype heat map. Mantel test was performed to infer the effect of geographic distance on genetic divergence. PLINK v1.9 was used to calculate pairwise genetic distance of identity by state (IBS) for each population [29]. The geographic distance matrix among each population was calculated in accordance with latitude and longitude. Finally, the correlation between geographic distance and genetic distance was accomplished in R v3.4.3.

## Results

### Phylogenetic inference and genetic structure

**Fig. 1:**
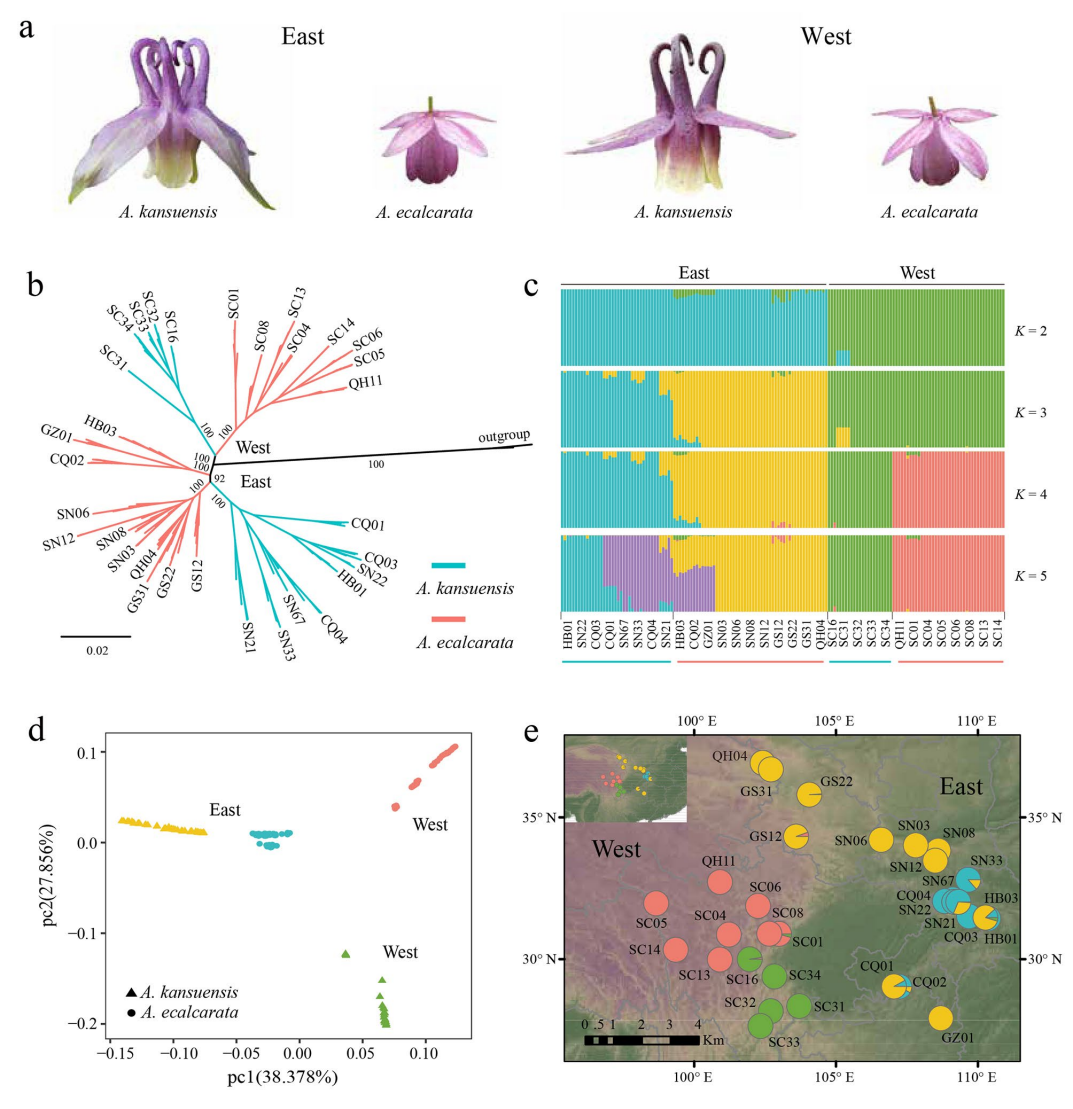
Phylogenetic inference and genetic structure. **(a)** Floral morphology of two species pairs (Eastern species pair and Western species pair). **(b)** The Maximum Likelihood (ML) phylogenetic tree of 158 individuals of *A. kansuensis* and *A. ecalcarata*. **(c)** Results from ADMIXTURE with *K* = 2, 3, 4, 5 based on SNP data. **(d)** The first two dimensions of principal component analysis (PCA) based on SNP data. **(e)** Geographic distribution of the sampling locations. The colors represent ancestral genetic components (according to ADMIXTURE at *K* = 4)

The ML phylogenetic tree was performed based on all SNPs (Fig. 1b). The phylogenetic tree was divided into two distinct clades with high bootstrap support. According to the geographical distribution of *A. kansuensis* and *A. ecalcarata* (Fig. 1e), we further called the two large clades as the Western group and the Eastern group (corresponding to Western species pair and Eastern species pair). ADMIXTURE was performed to analyze population genetic structure, When *K* = 2, the Eastern group and Western group had specific genetic components. When *K* = 3, the populations in the Eastern group demonstrated two major genetic components. Meanwhile, the genetically mixed populations (HB03, GZ01 and CQ02) appeared, which were more pronounced at *K* = 4 (Fig. 1c and e). And the Western group simultaneously showed two genetic components corresponding to *A. kansuensis* (SC16, SC31, SC32, SC33 and SC34) and *A. ecalcarata* (QH11, SC01, SC04, SC05, SC06, SC08, SC13 and SC14). When *K* = 5, part of *A. kansuensis* in the Eastern group had a new genetic component. The results of PCA of all SNPs also reflected a population genetic structure (Fig. 1d). The first principal component (pc1; variance explained = 38.378%) clearly separated the Western group and the Eastern group. And the second principal component (pc2; variance explained = 27.856%) clearly separated *A. kansuensis* and *A. ecalcarata* in Western group.

### Demographic history and gene flow

**Fig. 2:**
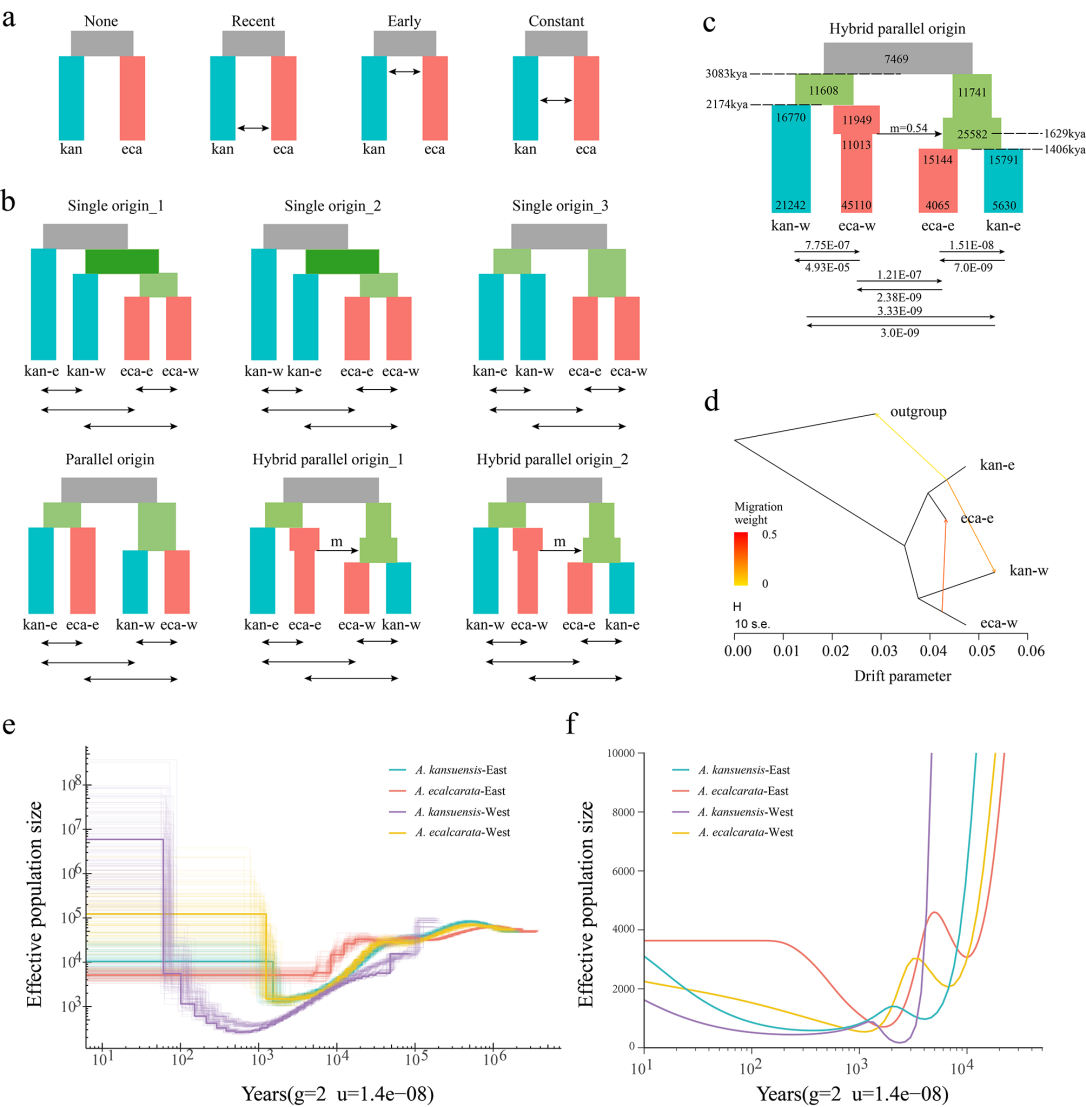
Demographic history and gene flow. **(a)** Four different models for inferring the gene flow patterns between species pair. **(b)** Comparing demographic models with different topologies (single, parallel and hybrid origin) with recent gene flow scenarios. A black arrow with ancestry proportion m indicates introgression event. Other black arrows indicate gene flow. **(c)** The demographic model with the best fit. Rectangles represent populations, whereas the numbers inside the rectangle indicates effective population size and the numbers corresponding to the dotted lines indicate the splitting times. **(d)** Three gene flow events inferred by Treemix. kan-w: Western *A. kansuensis*; eca-w: Western *A. ecalcarata*; kan-e: Eastern *A. kansuensis*; eca-e: Eastern *A. ecalcarata*. **(e)** and **(f)** PSMC and SMC + + estimations of the effective population size (Ne) for Eastern and Western species pairs. The time scale on the x-axis is calculated assuming a neutral mutation rate (µ = 1.4e-08 per site per year) and generation time (g = 2 years)

The highest likelihood distribution and the lowest AIC of Eastern and Western species pairs was both obtained for a model of divergence with recent gene flow (Fig. 2a; Figure S1 and S2). Among the six models for the origin of *A. ecalcarata*, the hybrid parallel origin model of the Eastern species pair derived from hybrid ancestors was the best model (highest likelihood distribution and lowest AIC) (Fig. 2c and S3; Table S3 and S4). Under this model, the first divergent event into *A. kansuensis* and *A. ecalcarata* in the West occurred at 2,174 kya (95% CI: 2,150-2,198 kya).

Subsequently, *A. ecalcarata* colonized the East and introgressed into the local *A. kansuensis* population (95% CI: 1,610-1,648 kya). Shortly after the introgression event, the admixed population was split into two population at 1,406 kya (95% CI: 1,394-1,418 kya). It was estimated that this introgression event was estimated to contribute a high proportion of the genetic variation (54%) of the ancestor of both Eastern species. Gene flow between the Western species pair was higher than that between the Eastern species pair, while recent gene flow was especially high from Western *A. ecalcarata* into Western *A. kansuensis*. To further verify the results of fastsimcoal2, Migrate-n software were used to infer gene flow between the Eastern and Western *A. ecalcarata*. The results indicate that Model 6 has the highest likelihood and probability (Table S5), supporting the hypothesis that Western *A. ecalcarata* had a past gene flow to the ancestral population of Eastern group.

PSMC and SMC + + were also used to infer population demographic (Fig. 2e and f). The results of PSMC analysis indicated a decrease in the *Ne* (effective population size) of Eastern and Western species pairs before 2 kya (kilo year ago), and the impact is greatest in Western *A. kansuensis.* After that, different groups underwent varying degrees of population expansion. SMC + + analysis also showed that four groups experienced different degrees of population contraction and expansion ranging from 1 to 10 kya, and Western *A. kansuensis* was more affected than Western *A. kansuensis*, and the same pattern was also observed in *A. ecalcarata*. These results suggest that the western population may have been more severely affected by the Quaternary glacial period.

**Fig. 3:**
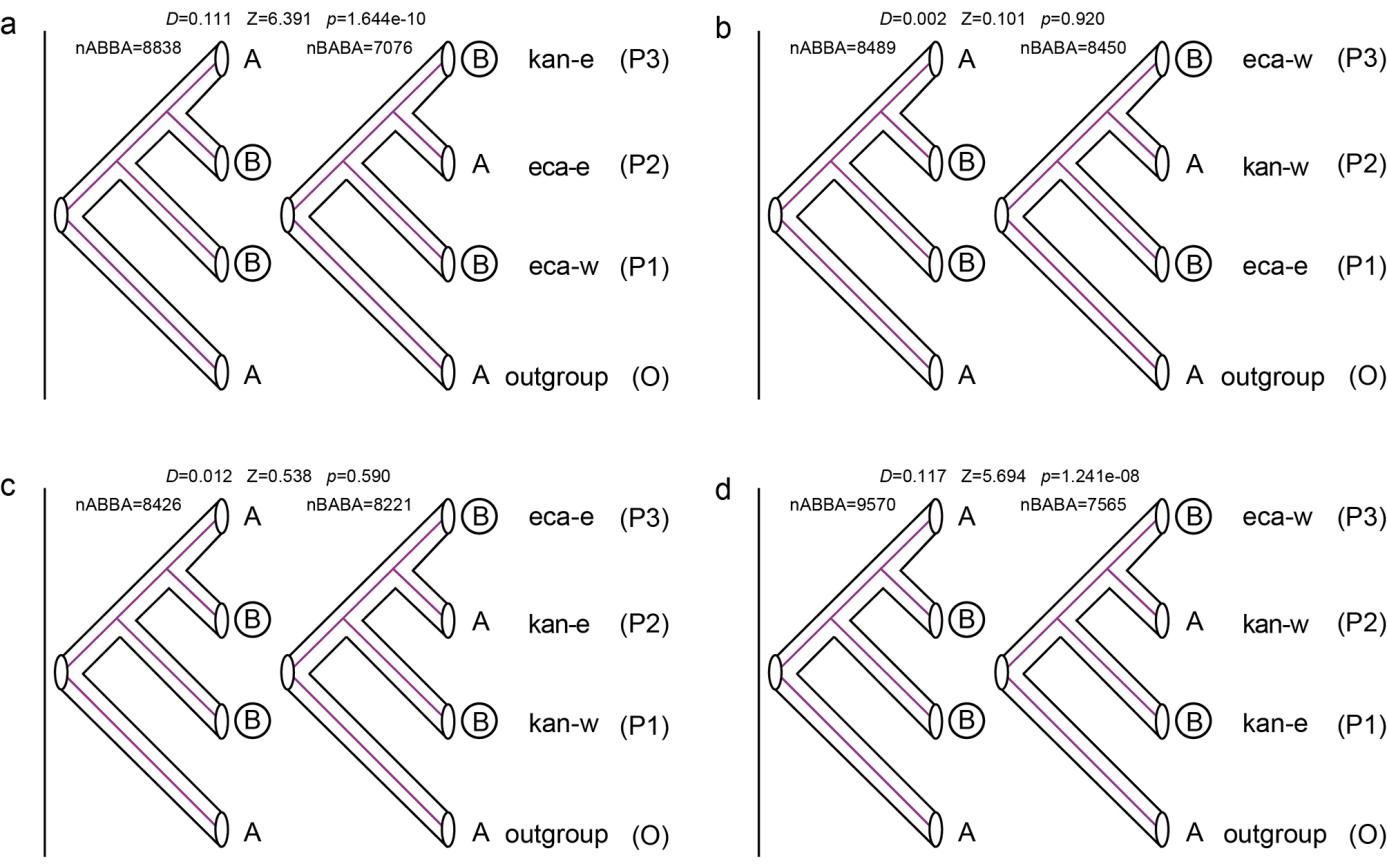
Genetic introgression inference. **(a-d)** Analysis of ABBA-BABA. A non-zero *D* statistic reflects an asymmetric pattern of allele sharing, implying gene flow. Z value and *p* value reflect the significance of the test. An absolute value of the Z score above 3 is often used as a critical value

The potential intraspecific and interspecific gene flow were also examined with Treemix and *D*-statistic. The OptM determined the optimal migration model as m = 3, suggesting that three migration edges might have occurred (Fig. 2d and S4). A strong signal of gene flow was detected from Western *A. ecalcarata* to Eastern *A. ecalcarata*. Furthermore, two other relatively weak gene flow were inferred from Eastern *A. kansuensis* to Western *A. kansuensis* and outgroup. The result was confirmed by the remarkable D value in *D*-statistic (Fig. 3). Gene flow between Eastern *A. ecalcarata* and Western *A. ecalcarata* had occurred as well when Eastern *A. kansuensis* was P1 (Fig. 3a), while the D value (0.111) was remarkable (*p* = 1.644e-10), and *f*4 admixture ratio (*f*4-ratio) was 9.68% (Table 1). Another significant (*p* = 1.241e-08) gene flow between Western *A. kansuensis* and Eastern *A. kansuensis* (0.117) had occurred when Western *A. ecalcarata* was P1 (Fig. 3d), and *f*4 admixture ratio (f4-ratio) was 8.83% (Table 1).

**Table 1:** The results of D-statistic and f4-ratio among the four Trios

P1	P2	P3	D-statistic	Z-score	*p*-value	f4-ratio	ABBA	BABA
kan_east	eca_east	eca_west	0.111	6.391	1.64E-10	0.0968	8838	7076
eca_west	kan_west	eca_east	0.002	0.101	0.92	0.0039	8489	8450
eca_east	kan_east	kan_west	0.012	0.538	0.59	0.0066	8426	8221
eca_west	kan_west	kan_east	0.117	5.694	1.24E-08	0.0883	9570	7565

### Shared high differentiation regions between species pairs

**Fig. 4:**
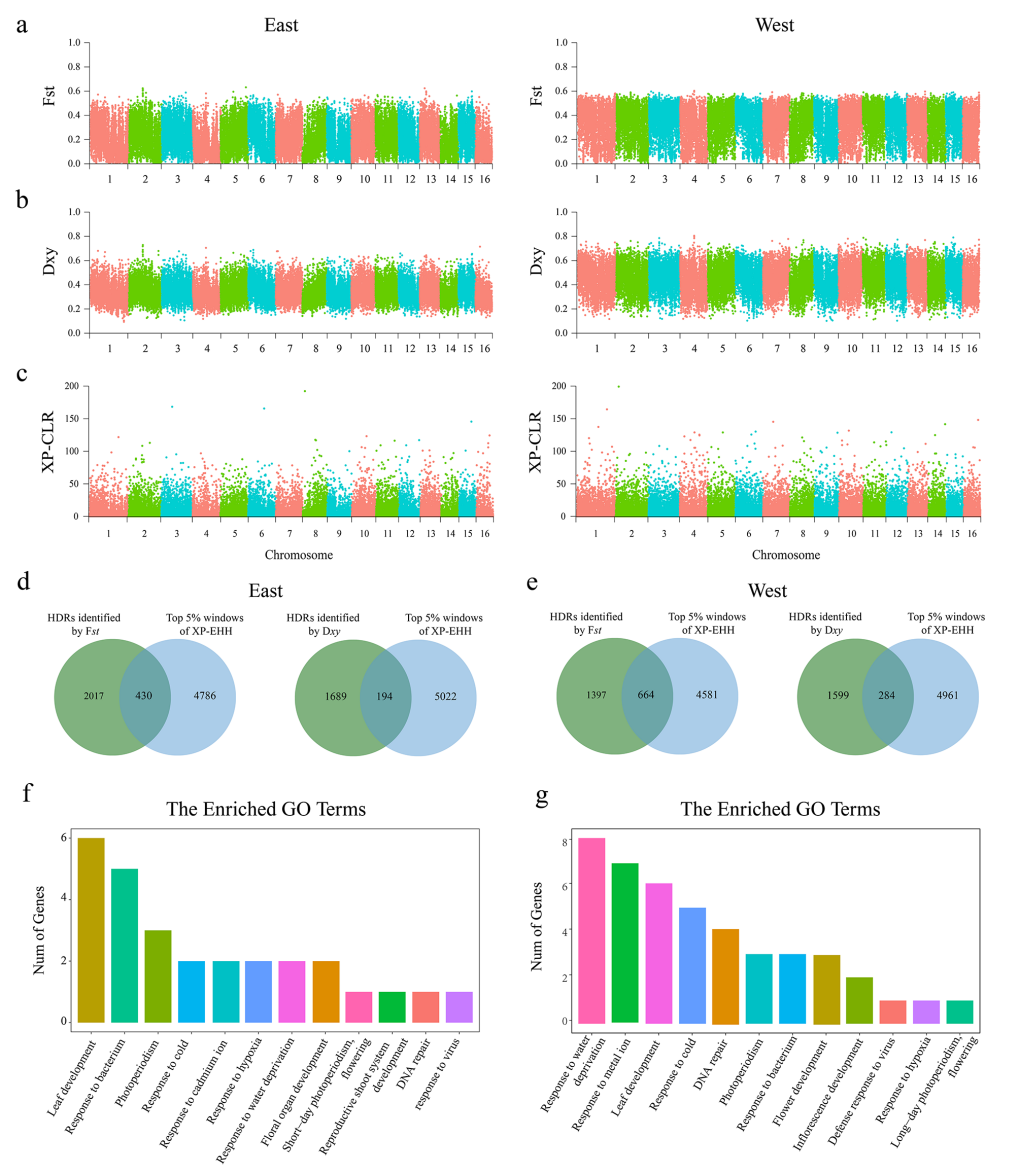
The genetic divergence and selection pressure among Eastern and Western species pairs. **(a)** The relative sequence divergence among species pairs. **(b)** The absolute sequence divergence among species pairs. **(c)** Selective sweeps analysis based on XP-CLR. **(d)** and **(e)** Venn diagram plots with overlapping windows among HDRs and top 5% XP-EHH. **(f)** Enrichment categories of candidate positive selection region based on XP-EHH in Eastern *A. ecalcarata*. **(g)** Enrichment categories of candidate positive selection region based on XP-EHH in Western *A. ecalcarata*

To determine the parallelism and non-parallelism of genetic divergence, a sliding window was used to calculate the genetic divergence (*Fst*) among the species pairs (Fig. 4a), and windows with Z-*Fst* ≥ 2 or Z-*Dxy* ≥ 2 were identified as highly diverged regions (HDRs). This approach resulted in 2446 HDRs in Eastern species pair and 2061 HDRs in Western species pair based on Z-*Fst* ≥ 2, respectively. The *Fst* estimates for both species pairs were significantly higher in HDRs than in Non-HDRs (Figure S5a and S5b). By comparing the parts of the HDRs that overlap between Eastern species pair and Western species pair, we obtained 123 shared HDRs, accounting for 5.03% HDRs in Eastern species pair and 5.97% HDRs in Western species pair (Figure S6a). The sharing ratio of HDRs was significantly lower than that of Non-HDRs (Chi-square test, *p*-value = 2.2e-15). These results indicate that only a subset of the highly differentiated regions of the original species pair is also differentiated between the younger species, and most regions of the genome were non-parallel. The *Fst* estimates for both Eastern and Western species pairs were not significantly higher in shared HDRs than in remaining HDRs (Figure S7a and S7b). Nucleotide polymorphisms can substantially affect relative divergence (*Fst*), so we analyzed absolute sequence divergence (*Dxy*) (Fig. 4b). According to Z-*Dxy* ≥ 2, 1771 HDRs in Eastern species pair and 1741 HDRs in Western species pair were identified (Fig. 4d and e). The *Dxy* estimates for both species pairs were significantly higher in HDRs than in Non-HDRs (Figure S5c and S5d). By comparing the parts of the HDRs that overlap between Eastern species pair and Western species pair, we obtained 742 shared HDRs, accounting for 41.90% HDRs in Eastern species pair and 42.62% HDRs in Western species pair (Figure S6b). The sharing ratio of HDRs was significantly lower than that of Non-HDRs (Chi-square test, *p*-value = 2.2e-14). The *Dxy* estimates for both Eastern and Western species pairs were significantly higher in shared HDRs than in remaining HDRs (Figure S7c and S7d). These results indicate that the shared HDRs play an important role in the divergence of the species pair.

### Analysis of selection pressure

The selection pressure was performed through XP-CLR and XP-EHH analysis, and the candidate HDRs located in the top 5% XP-CLR or top 5% XP-EHH windows were considered as a candidate positive selection region. According to *Fst* and XP-CLR, a total of 930 HDRs had experienced positive selection in the Eastern species pair, accounting for 38% of the HDRs, and 351 HDRs had experienced positive selection in the Western species pair, accounting for 17% (Figure S8). According to *Dxy* and XP-CLR, a total of 82 HDRs had experienced positive selection in the Eastern species pair, accounting for 5% of the HDRs, and 142 HDRs had experienced positive selection in the Western species pair, accounting for 8% (Figure S8). Upon GO enrichment analysis of the candidate positive selection regions, we found that both Eastern and Western species were enriched in the category of regulation of photoperiodism, flowering, regulation of flower development, leaf development, seed germination, response to cold, DNA repair, defense response to bacterium (Figure S8; Gene IDs are listed in Table S6-S9 ).

According to *Fst* and XP-EHH, a total of 430 HDRs had experienced positive selection in the Eastern *A. ecalcarata*, accounting for 18% of the HDRs, and 664 HDRs had experienced positive selection in the Western *A. ecalcarata*, accounting for 32% (Fig. 4). According to *Dxy* and XP-CLR, a total of 194 HDRs had experienced positive selection in the Eastern *A. ecalcarata*, accounting for 11% of the HDRs, and 284 HDRs had experienced positive selection in the Western *A. ecalcarata*, accounting for 16% (Fig. 4). Upon GO enrichment analysis of the candidate positive selection regions, we found that both Eastern and Western species were enriched in the category of response to water deprivation, response to hypoxia, regulation of photoperiodism, flowering, regulation of flower development, leaf development, seed germination, response to cold, DNA repair, defense response to bacterium and virus (Fig. 4; Gene IDs are listed in Table S10-S13).

This suggests that the photoperiodism, precipitation, temperature, DNA repair, flowering time, regulation of flower and leaf development, seed germination, and defensive response of bacterium and virus may be important driving factors behind the parallel evolution of eastern and western *A. ecalcarata*.

### Gene flow in highly diverged regions

**Fig. 5:**
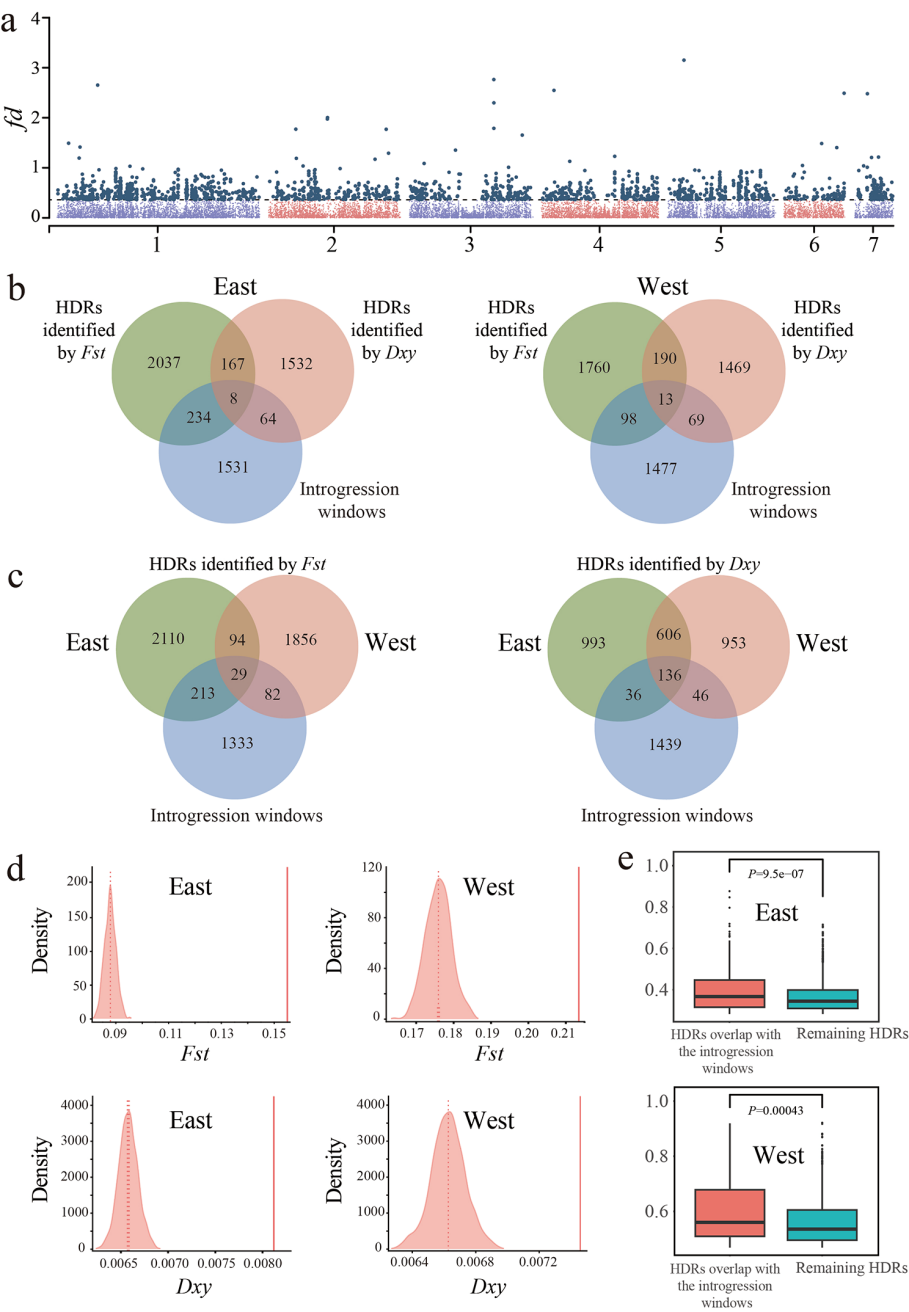
The influence of gene flow on the divergence of species pairs. **(a)** Manhattan plot showing the *fd* values across genome. Dark spots indicate the locations of the candidate introgressed regions. **(b)** Venn diagram plots with overlapping windows between HDRs and Introgression. **(c)** Venn diagram plots with overlapping windows between shared HDRs and Introgression. **(d)** Diagram showing the comparison of genetic divergence (*Fst* and *Dxy*) between introgressed regions (solid line) and the distribution resulting from the 1,000 randomizations (dashed line: average across randomizations). **(e)** The *Fst* distribution for both species pairs were significantly higher in the HDRs overlap with the introgression windows than in remaining HDRs

Determining whether the phenotypic similarity between two similar taxa stems from hybridization is a major challenge because the magnitude of gene flow can vary among regions in the genome. We detected gene flow in the parallel diverged regions of the Eastern and Western species pairs. According to *D*-statistical analysis, the trio (kan_east, eca_east), eca_west) revealed gene flow between Eastern *A. ecalcarata* and Western *A. ecalcarata* had occurred (Fig. 3a), and *f*4 admixture ratio (*f*4-ratio) was 9.68% (Table 1). So we further localized introgressed regions by calculating *fd* statistics, which have been proven to be more useful to assist in locating introgressed loci in small genomic regions compared with the *D*-statistics. Introgressed regions were defined as the top *fd* windows that summed to the genomic proportion estimated from the *f*4-ratio (9.68%), and 1657 introgression windows were identified (Fig. 5a). According to *Fst*, 242 HDRs in Eastern species pairs overlap with the introgression windows, which accounted for 10% of the HDRs (2446 HDRs), and 111 HDRs in Western species pairs overlaps with the introgression windows, which accounted for 5% of the HDRs (2061 HDRs) (Fig. 5b). 24% (29/123) shared HDRs overlap with the introgression windows between Eastern and Western species pairs (Fig. 5c). According to *Dxy*, 72 HDRs in Eastern species pairs overlap with the introgression windows, which accounted for 4% of the HDRs (1771 HDRs) in the Eastern species pairs, and 82 HDRs in Western species pairs overlaps with the.

introgression windows, which accounted for 5% of the HDRs (1741 HDRs) in the Western species pairs (Fig. 5b). 5% (29/123) shared HDRs overlap with the introgression windows between Eastern and Western species pairs (Fig. 5c). The proportion of both shared HDRs by *Fst* and *Dxy* overlap with the introgression windows is significantly higher than that of the remaining HDRs (Chi-square test, *p*-value = 2.2e-16; Chi-square test, *p*-value = 0.0028). These results indicate that gene introgression plays a very important role in the parallel evolution of Eastern and Western *A. ecalcarata*.

To further demonstrate the impact of gene introgression on genetic divergence in Eastern and Western species, we compared the genetic divergence coefficients between the introgression window and the 1000 random sampling windows. The *Fst* and *Dxy* estimates of introgression windows for both species pairs were significantly higher than at random (Fig. 5d). The *Fst* estimates for both species pairs were significantly higher in the HDRs overlap with the introgression windows than in remaining HDRs (Fig. 5e). Both model analysis and gene flow detection showed that there was a significant gene flow from western to eastern species, so these results reflect the significant effect of the gene flow from Western *A. ecalcarata* to Eastern *A. ecalcarata* on the origin and divergence of the Eastern species pair.

### Gene flow and function in shared positive selection regions

**Fig. 6:**
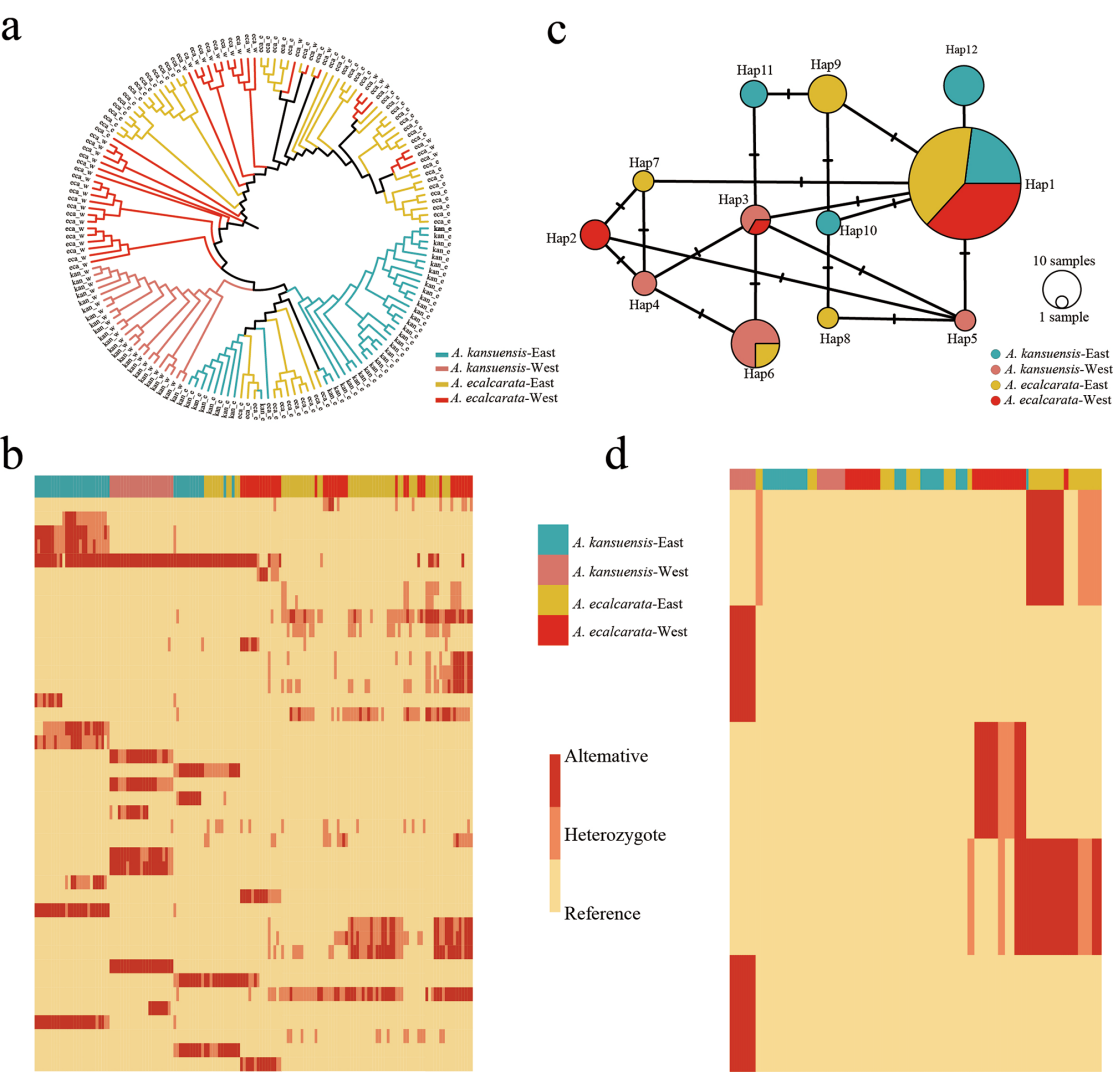
Phylogeny and haplotype analysis of *PIA2* and *POPVICH* genes. **(a)** The ML phylogenetic tree of *PIA2* gene in all individuals of *A. ecalcarate* and *A. kansuensis*, Jones-Taylor-Thornton model. **(b)** Heatmap analysis of *PIA2* gene. Each row represents a genomic position for all accession, and the column represents a individual. (**c)** Median-joining network of *POPVICH* gene haplotypes. The areas of the circles are proportional to the number of individuals. **(d)** Heatmap analysis of *POPVICH* gene. Each row represents a genomic position for all accession, and the column represents a individual

Based on the XP-CLR and *Fst*, Eastern and Western species share 8 candidate positive selection HDRs, four of which have higher gene flow, including two genes. Based on the XP-CLR and *Dxy*, Eastern and Western species share 4 candidate positive selection HDRs, all of which have higher gene flow, including 3 genes. By conducting GO analysis on these genes, the category of proton-transporting ATP synthase activity, G-protein coupled receptor activity, protein binding were enriched. (GO Terms and Gene IDs are listed in Table S14). Based on the XP-EHH, there are no shared HDRs with higher gene flow. In order to further clarify the functions of these gene IDs, we conducted gene identification, and found that the gene corresponding to Chr1_20.1976 is named *PIA2*, which has a function of response to high light intensity [43]. The phylogenetic tree and heat map show that the *PIA2* gene has higher similarity between Eastern and Western *A. ecalcarata* (Fig. 6a and b).

we also analyzed the haplotypes of *POPOVICH*. A total of 12 haplotypes were generated from 158 individuals (Fig. 6b). Haplotype network and heatmap cluster tree showed that *POPOVICH* did not differentiate between *A. kansuensis* and *A. ecalcarata*, and there was no pattern of two major branches in the East and West (Fig. 6c and d). The haplotype network revealed that *A. kansuensis* and *A. ecalcarata* shared many haplotypes. Moreover, The window chromosome3_26779918–26,781,011 in which *POPOVICH* was located did not belong to the HDRs, and the *fd* values of this window did not rank in the top 9.68% of the entire genome.

### Isolation by distance (IBD)

In the Western species pair, a Mantel test revealed that genetic distance and geographic distance were significantly correlated (i.e., isolation by distance) between *A. ecalcarata* and *A. kansuensis*. A significant pattern of IBD was also detected between *A. ecalcarata* and *A. kansuensis* in the Eastern species pair. We also combined the Eastern and Western groups for IBD analysis, and the results still showed a significant relationship (Table S15). These findings suggested that geographical isolation contributes to the genetic divergence between *A. ecalcarata* and *A. kansuensis.* The value of the Mantel statistic in the Western species pair was higher than that in the Eastern species pair, indicating that geographical isolation had a greater effect on genetic differentiation in the Western species pair than in the Eastern species pair.

## Discussion

### Parallel origin of *Aquilegia ecalcarata* after hybridization

Systems with either parallel evolution or parallel maintenance of species differences are thus both useful for studying the processes underlying natural selection in the evolution of species or traits [12, 14]. It is crucial to study the underlying evolutionary mechanisms of parallel evolution, including natural selection, de novo mutations, gene flow, drift and standing genetic variation [14, 44]. However, tests discriminating single and multiple origins of ecotypes or species in the face of persistent gene flow are often lacking. Based on population level sampling, Huang et al. [45] found that *A. ecalcarata* is not monophyletic. But due to lack of resolution, the origin of *A. ecalcarata* and its phylogenetic relationships with related species remain unclear. Geng et al. [19] used genomic data to analyze the origin of *A. ecalcarata*, and the results indicated that *A. ecalcarata* could be divided into different groups; however, whether the different groups of *A. ecalcarata* were derived from independent parallel origins or genetic introgression remains unclear. In this study, demographic model showed that the genetic differentiation first occurred between *A. ecalcarata* and *A. kansuensis* in the Western group. Then *A. ecalcarata* in the Western group colonized the East and hybridization with the ancestral population in Eastern *A. kansuensis.* Western *A. ecalcarata* contributed 54% of the genetic components to the hybrid progeny, while Eastern ancestral population contributed 46% to the hybrid progeny, which is similar to the genetic contribution ratio of many hybrid cases, and the parents is close to 50% [14, 46, 47]. Shortly after the hybridization event, the admixed population was split into *A. ecalcarata* and *A. kansuensis* in the East. Analysis of Treemix and *D*-statistic also revealed that a strong signal of gene flow was detected from Western *A. ecalcarata* to Eastern *A. ecalcarata*. Thus, the origin of Western *A. ecalcarata* preceded the hybrid origin of Eastern *A. ecalcarata*. This pattern of parallel origin of *A. ecalcarata* species after hybridization is consistent with the hybrid parallel origin model of *Pundamilia cichlid* species [14, 16]. Parallel origin in the narrow sense emphasizes the independence of the evolutionary history of the two independently evolving pairs [48], and some well-known cases have been documented in sticklebacks [49], stick insects [17], wildflowers [50] and wild rice [51]. Hybrid parallel origin emphasizes the key role of introgression or hybridization in the divergence of repetitive evolving pairs of ecotypes or species. However, hybrid parallel origin events has been rarely reported. The results of our study provide new evidence for hybrid parallel origin of ecotypes or species.

On the other hand, the results from TreeMix and *D*-statistic conflict with the “best” model in Fig. 2c. The introgression from Western *A. ecalcarata* to the ancestry of Eastern species pair, so the migration edge to point to the internal branch instead of the tip branch of Eastern *A. ecalcarata*. similarly, the *D* value in Fig. 3a should not be significant because both the Eastern *A. ecalcarata* and *A. kansuensis* inherited the same introgression that occurred in ancestry. The reason for this conflict is that both TreeMix and *D* values measured the results of gene flow, reflecting only the results of past hybridization. The ancestral population in the Eastern population with spur crossed with Western *A. ecalcarata* to produce the Eastern *A. ecalcarata* and *A. kansuensis*. However, due to the adaptability of the Eastern population to the local climate, the Eastern *A. ecalcarata* and *A. kansuensis* will continue to backcross with eastern ancestral population to adapt to the local environment, and the phenotype of the Eastern *A. kansuensis* and Eastern ancestors with spur are more similar, the phenotype and habitat of the eastern and western *A. ecalcarata* are more similar, so there was more subsequent gene flow from the Western *A. ecalcarata* lineage to the Eastern *A. ecalcarata* lineage. Therefore, Eastern *A. ecalcarata* and *A. kansuensis* are unlikely to inherit the same infiltration that occurred in the ancestors. The simulation of the model is a simulation of past gene flow events, highlighting the process rather than the result, so it is different from the results of TreeMix and *D*-statistic.

### Ecological adaptation and genetic mechanism underlying the hybrid parallel origin of *A. Ecalcarata*

Natural selection is an important driving force for parallel evolution [2, 3]. Huang et al. [18] described that *A. ecalcarata* and *A. kansuensis* have different habitats. *A. kansuensis* grows in fertile soil under low altitude forests, while *A. ecalcarata* grows on stony beaches with poor soil at high altitude, habitat shift may be an important driving factor in the multiple origins of *A. ecalcarata*. Our analysis of genetic divergence also revealed that environmentally-related pathways such as response to water deprivation, response to hypoxia, regulation of photoperiodism, flowering, regulation of flower development, leaf development, seed germination, response to cold, DNA repair are important drivers of divergence among eastern and western species pair. Secondly, the *PIA2* gene is located in HDR shared by both Eastern and Western species pair with higher gene flow, and responds to high light intensity. Therefore, differences of the climate factors might have contributed to the divergence between *A. ecalcarata* and *A. kansuensis* and the parallel origins of *A. ecalcarata*. However, additional work is needed to verify these speculations.

The spurless trait is a novel phenotype of *A. ecalcarata* that has contributed to the divergence between *A. ecalcarata* and *A. kansuensis* [19]. Therefore, the key gene controlling the nectar spur is likely highly divergent between *A. ecalcarata* and *A. kansuensis*. Ballerini et al. [20] found that the *C2H2* transcription factor *POPOVICH* plays a key role in spur formation, but the window in which the *POPOVICH* gene was located (chromosome3_26779918–26,781,011) was not one of the HDRs (top 10% *Fst* windows) in the Eastern and Western species pairs. Phylogenetic tree showed that *POPOVICH* did not differentiate between *A. kansuensis* and *A. ecalcarata*. Therefore, the *POPOVICH* gene might not be the key candidate gene underlying divergence in the nectar spur in our species pairs. However, the extent to which *POPOVICH*’s expression level varies within our species remains unclear and is a question that needs to be explored in the future.

### Non-parallelism of the genomic differentiation between the independently evolving species pairs

Analysis of genomic differentiation revealed that 123 HDRs identified by *Fst* and 742 HDRs identified by *Dxy* were shared between Eastern and Western species pairs, the number of non-shared HDRs was 2323 (identified by *Fst*) and 1029 (identified by *Dxy*) in Eastern species pair, and the number of non-shared HDRs was 1938 (identified by *Fst*) and 999 (identified by *Dxy*) in Western species pair, which indicated that most of the HDRs in the genome were non-parallel. Thus, the genetic differentiation in the Eastern species pair was not restricted to a subset of the genomic differences that characterize the Western species pair; instead, genomic differentiation likely included several new regions in the Eastern species pair, which reflects the independence of the divergence among species pairs. Many factors might contribute to explaining the observed non-parallelism among species pairs. First, the differences in the divergence of phenotypic traits between Eastern and Western species pairs might explain non-parallelism, as previous studies have shown that the direction of differentiation in the three floral traits in Eastern and Western species pairs differs [19]. Second, demographic history of Eastern and Western species pairs might lead to differences in the divergence process. Because the sudden and large decrease in population size due to the bottleneck may affect population divergence, thereby increasing the possibility of fixing mildly deleterious and effectively neutral mutations [52–54]. Our demographic history indicated that both the Eastern and Western species pairs have experienced varying degrees of population contraction. Thus, the Eastern and Western species pair might have experienced a bottleneck at some point in its evolutionary history, increasing the probability of the fixation of mildly deleterious and effectively neutral mutations.

## Conclusions

Our study supports the gene flow contributed to the parallel evolution of *A. ecalcarata*. The results of gene flow test reflect the significant effect of the gene introgression from Western *A. ecalcarata* to Eastern *A. ecalcarata* on the origin and divergence of the Eastern species pair. These findings provide new evidence for parallel origin after hybridization as well as insights into the mechanisms underlying the parallel origins of species. In the next study, we will still need to conduct field experiments and molecular biology experiments to explore the ecological adaptation and genetic mechanism of the repeated origin of *A. ecalcarata*.

### Electronic supplementary material

Below is the link to the electronic supplementary material.

Supplementary Material 1

Supplementary Material 2

## Data Availability

The genomic data support the finding of this study have been desposited in the GenBank database under BioProject: PRJNA690975.

## Abbreviations

AIC: Akaike information criterion
Fst: Genetic differentiation index
Dxy: absolute sequence divergence index
HDRs: highly diverged regions
2D-SFS: two-dimensional joint SFS
ECM: expectation-conditional maximization
IBS: identity by state
IBD: Isolation by distance
