# Supplementary material for: Genomics of hybrid parallel origin in Aquilegia ecalcarata
Source: BMC Ecol Evol. 2024 Jun 6;24:75. doi: 10.1186/s12862-024-02266-7 (PMC11155106; doi:10.1186/s12862-024-02266-7)
Supplement: Supplementary file 1 — Supplementary Material 1 [file 12862_2024_2266_MOESM1_ESM.docx]

**Supplementary information**


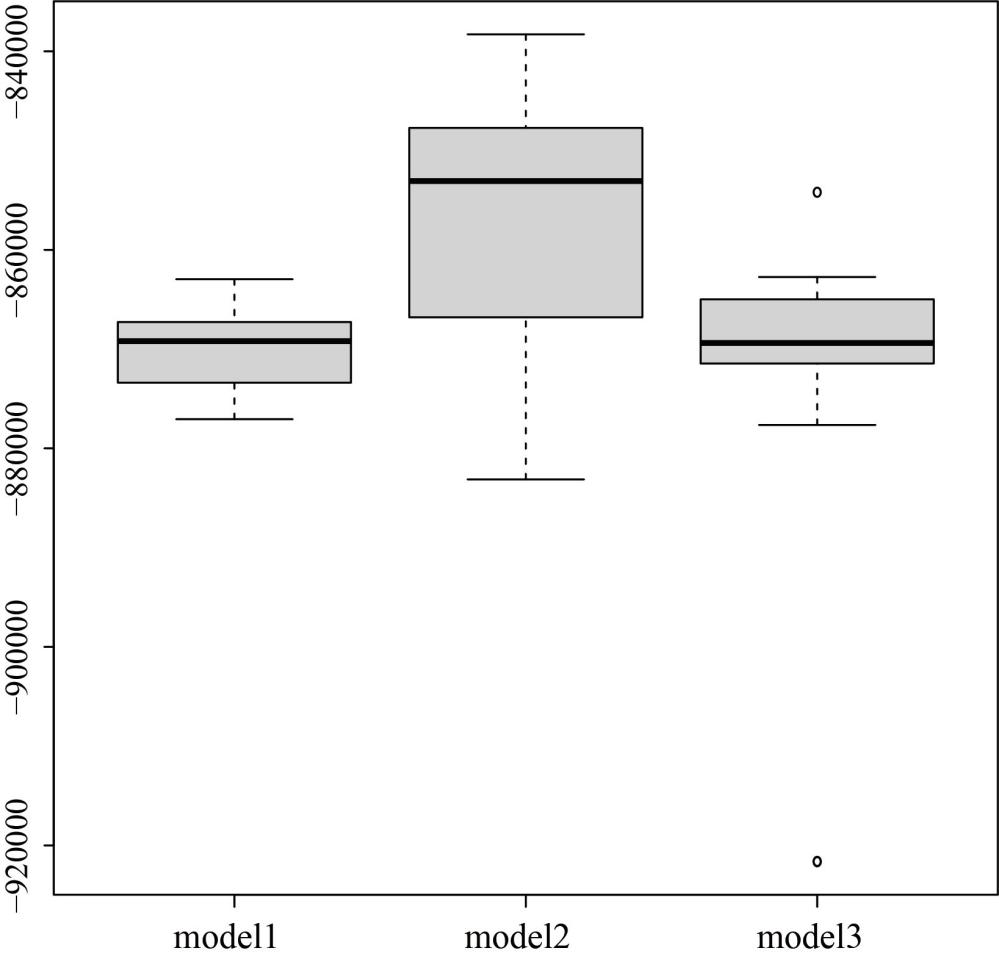


**Figure S1** Comparison of demographic models of eastern species pairs. Boxplots showing the log_10_ Likelihood distributions from 50 expected SFS each approximated using 100,000 coalescent simulations under the parameters that maximize the likelihood for each model.


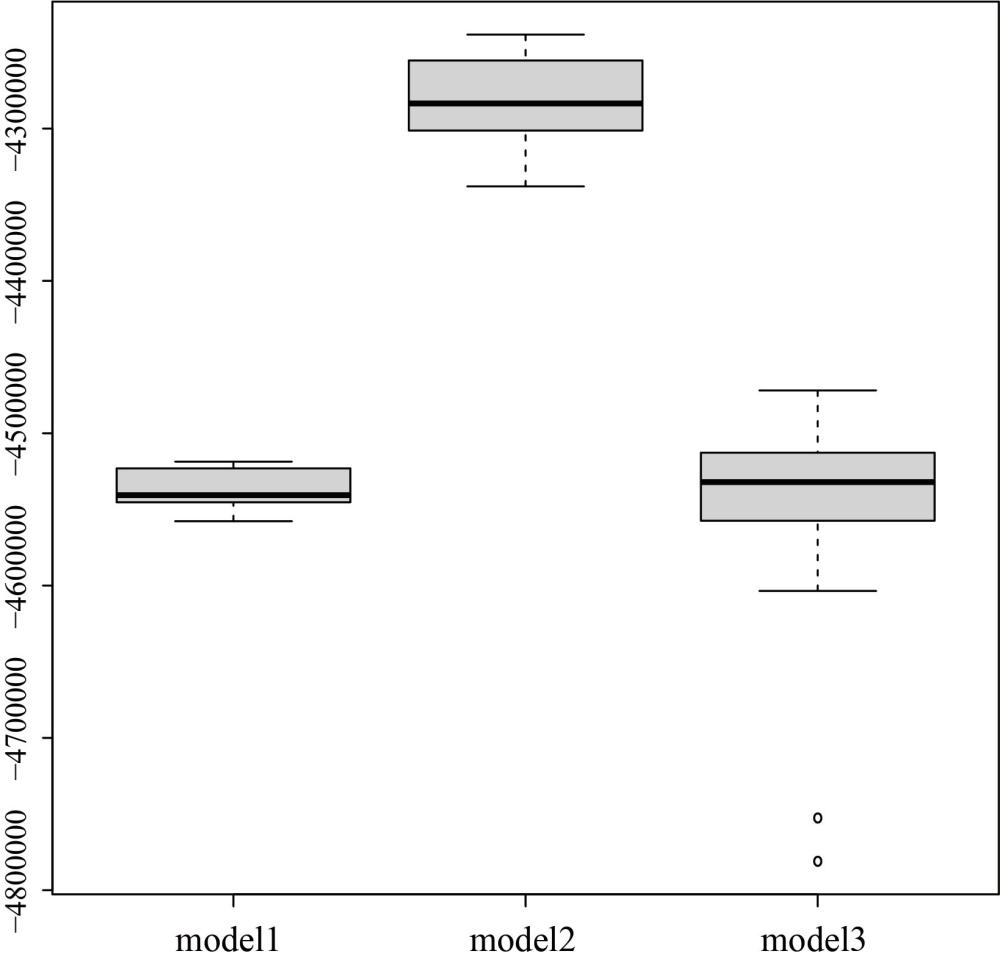


**Figure S2** Comparison of demographic models of western species pairs. Boxplots showing the log_10_ Likelihood distributions from 50 expected SFS each approximated using 100,000 coalescent simulations under the parameters that maximize the likelihood for each model.


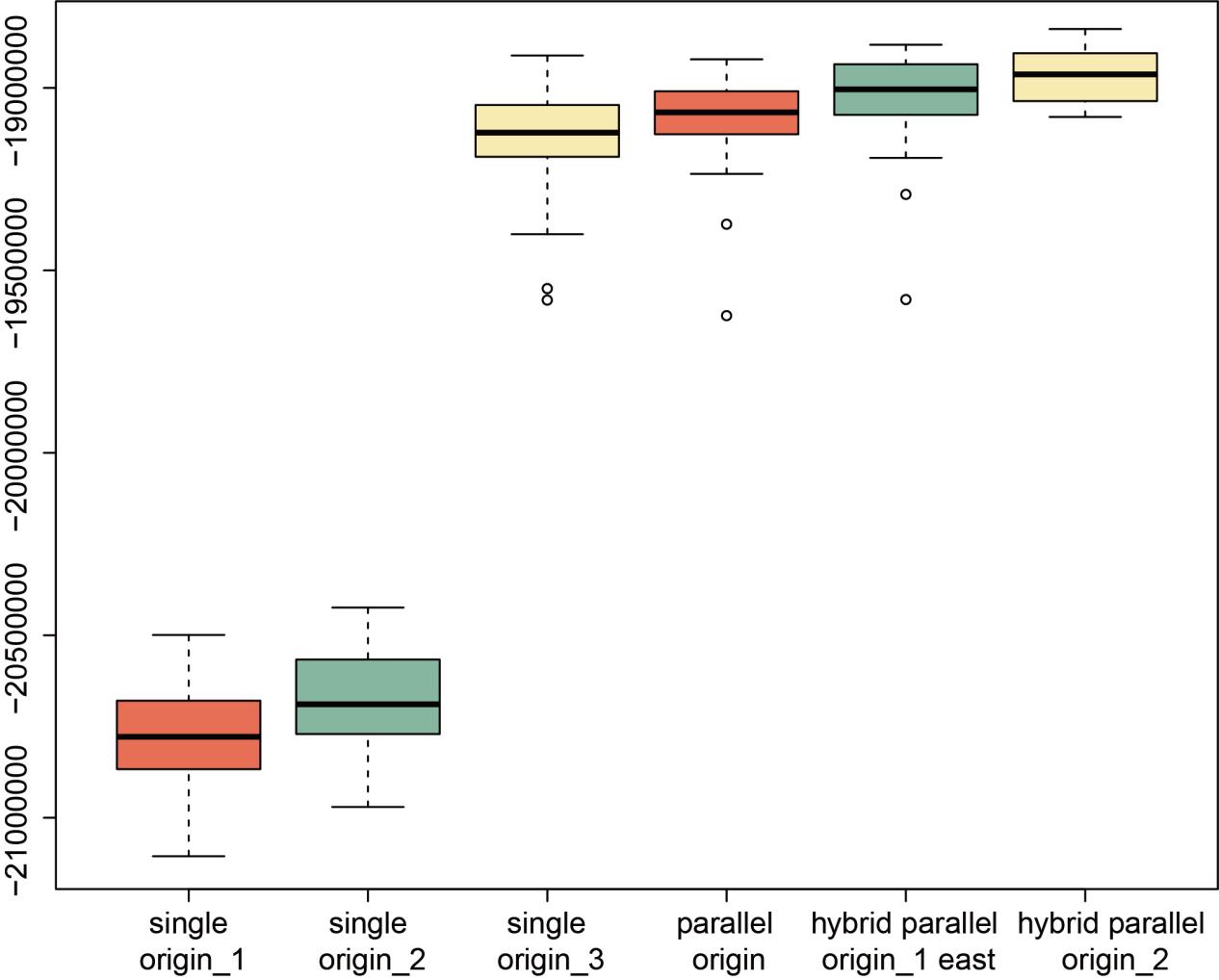


**Figure S3** Comparison of demographic models of pecies pairs. Boxplots showing the log_10_ Likelihood distributions from 50 expected SFS each approximated using 100,000 coalescent simulations under the parameters that maximize the likelihood for each model.

**
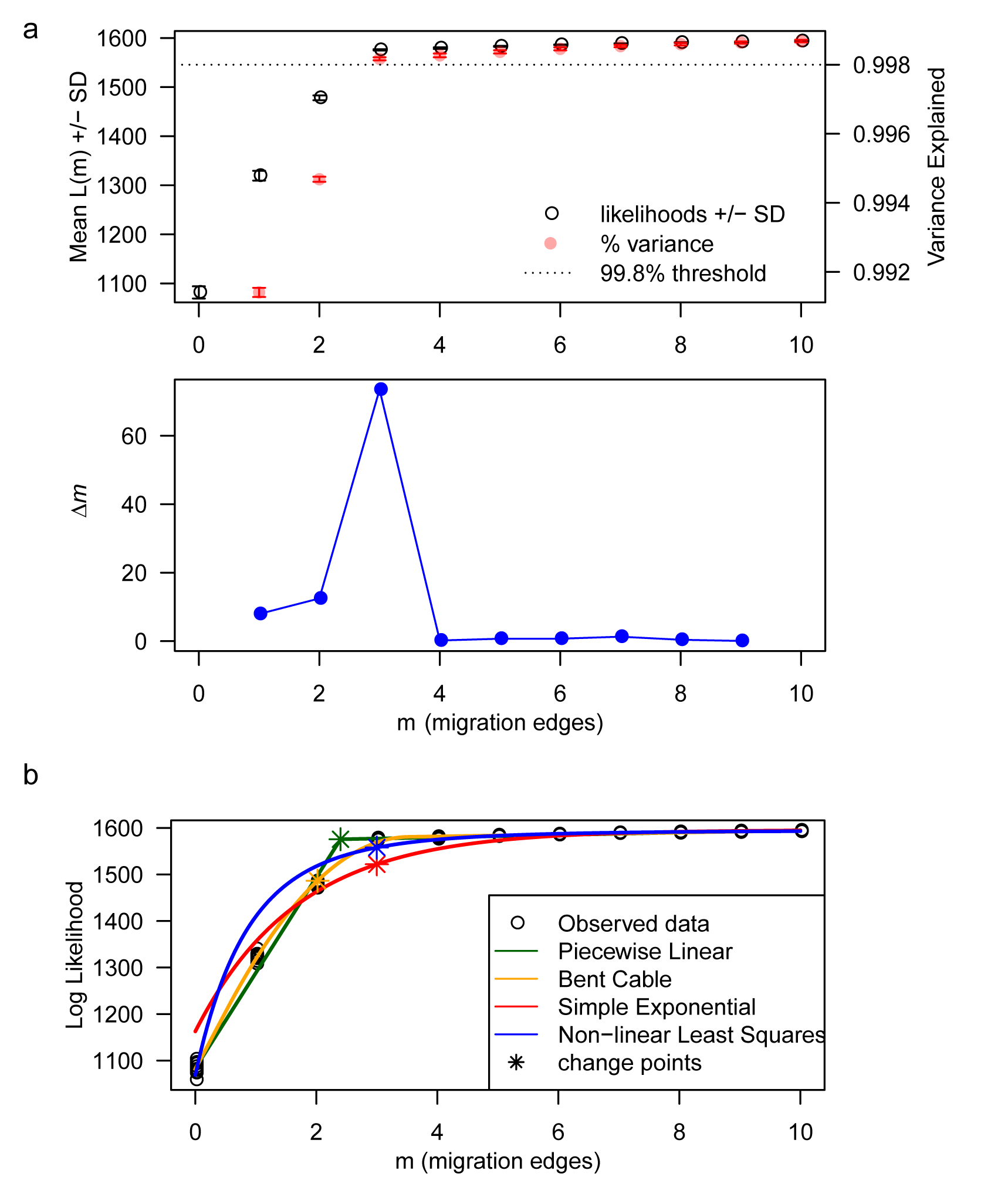
**

**Figure S4** Treemix models determining the best m value. (a) The default “Evanno”-like method in R package OptM. (b) The various linear model in R package OptM.

**
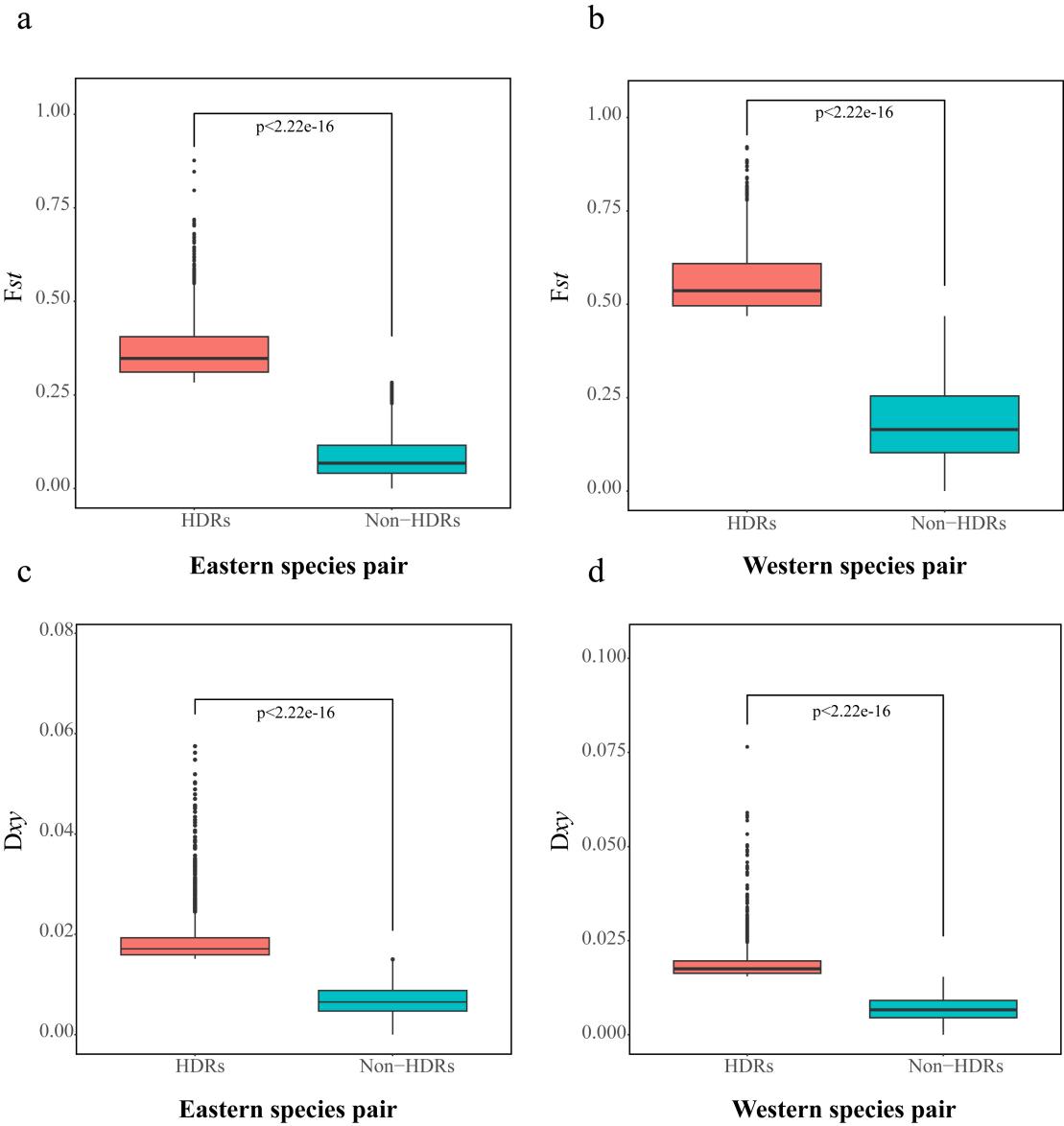
**

**Figure S5** Comparison of HDRs and Non-HDRs between species pairs. (a) Boxplots showing the distributions of *Fst* for the HDRs and Non-HDRs in Eastern species pair. (b) Boxplots showing the distributions of *Fst* for the HDRs and Non-HDRs in Western species pair. (c) Boxplots showing the distributions of *Dxy* for the HDRs and Non-HDRs in Eastern species pair. (d) Boxplots showing the distributions of *Dxy* for the HDRs and Non-HDRs in Eastern species pair.

**
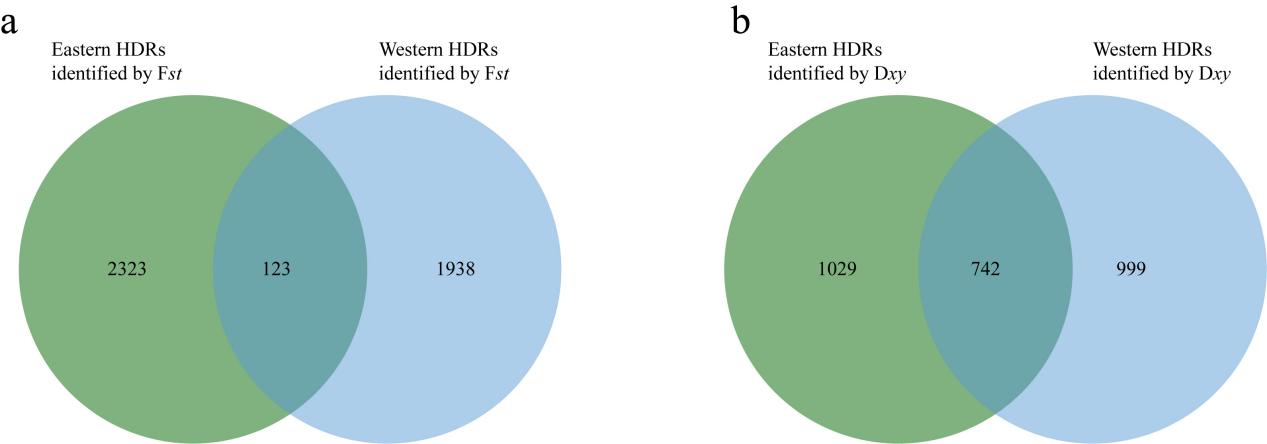
**

**Figure S6** Venn diagram plots with overlapping windows among HDRs. (a) Overlapping HDRs identified by *Fst* between Eastern and Western species pairs. (b) Overlapping HDRs identified by *Dxy* between Eastern and Western species pairs.


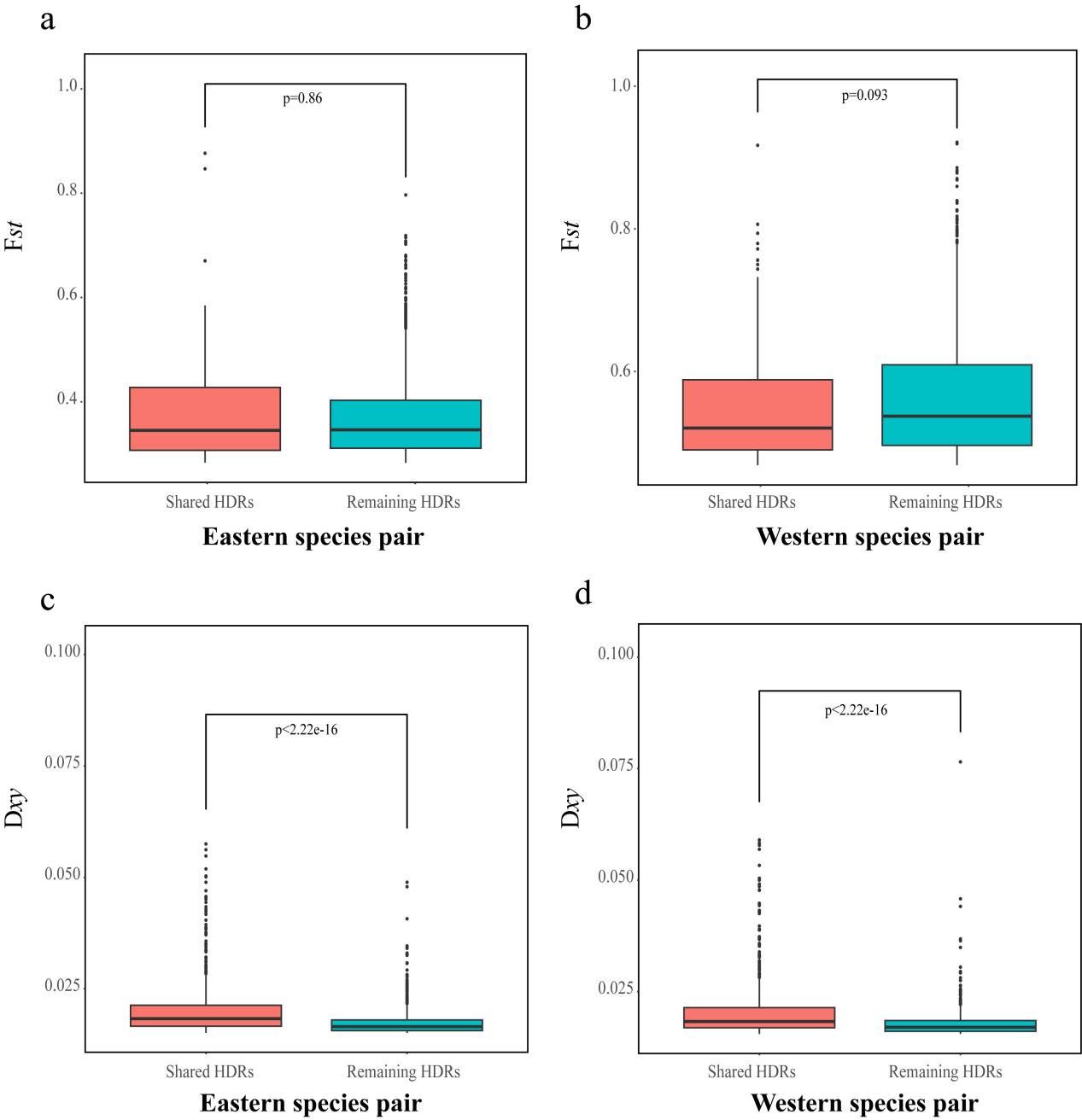


**Figure S7** Comparisons of HDRs between the species. (a) Boxplots showing the distributions of *Fst* for the shared HDRs and remaining HDRs in Eastern species pair. (b) Boxplots showing the distributions of *Fst* for the shared HDRs and remaining HDRs in Western species pair. (c) Boxplots showing the distributions of *Dxy* for the shared HDRs and remaining HDRs in Eastern species pair. (d) Boxplots showing the distributions of *Dxy* for the shared HDRs and remaining HDRs in Western species pair.


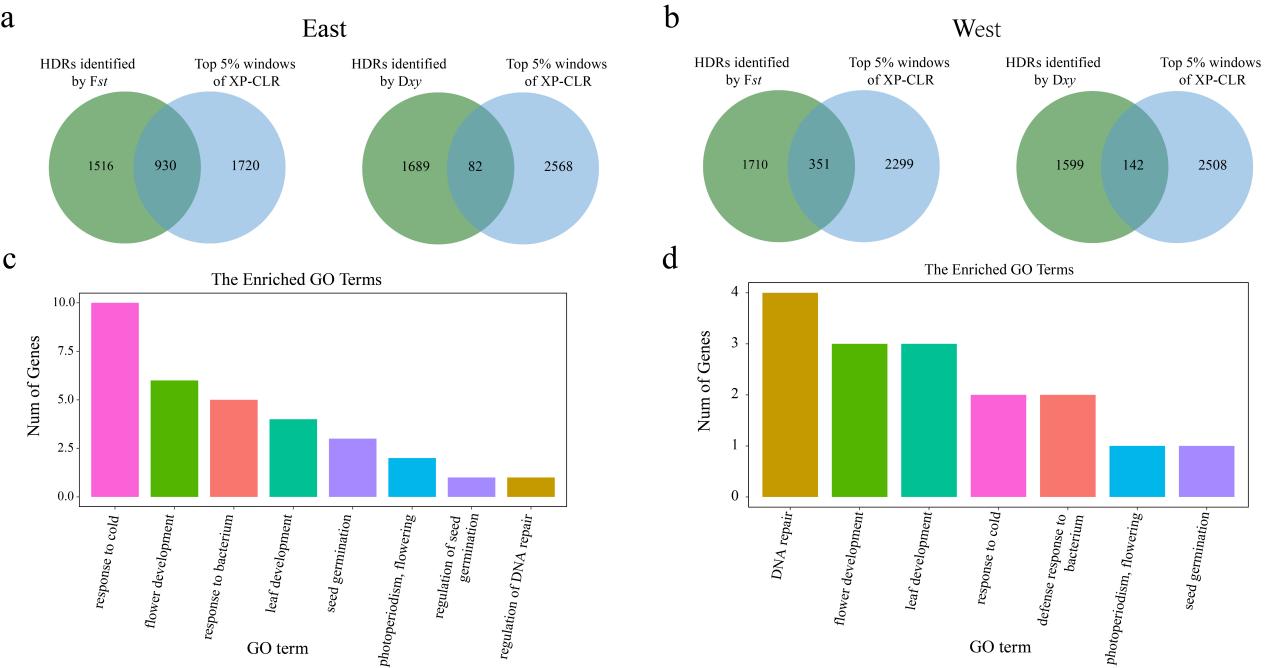


**Figure S8** Identification and GO enrichment analysis of the positive selection HDRs. (a) and (b) Venn diagram plots with overlapping windows among HDRs and top 5% XP-CLR. (c) Enrichment categories of candidate positive selection region based on XP-CLR in Eastern species pair. (d) Enrichment categories of candidate positive selection region based on XP-CLR in Western species pair.
